# Fe-Co Alloy Nanoparticles Dispersed in Polymer-Derived Carbon Support: Effect of Initial Polymer Nature on the Size, Structure and Magnetic Properties

**DOI:** 10.3390/ma16206694

**Published:** 2023-10-14

**Authors:** Andrey Vasilev, Mikhail Efimov, Dmitry Muratov, Petr Chernavskii, Kirill Cherednichenko, Ella Dzidziguri, Galina Karpacheva

**Affiliations:** 1 A.V. Topchiev Institute of Petrochemical Synthesis RAS, Leninskiy Prospekt 29, Moscow 119991, Russia; vasilev@ips.ac.ru (A.V.); efimov@ips.ac.ru (M.E.); muratov@ips.ac.ru (D.M.); chern5@inbox.ru (P.C.); 2 Department of Chemistry, Lomonosov Moscow State University, Leninskie Gory 1–3, Moscow 119991, Russia; 3 Department of Physical and Colloidal Chemistry, National University of Oil and Gas “Gubkin University”, Leninskiy Prospekt 65, Moscow 119991, Russia; cherednichenko.k@gubkin.ru; 4 Department of Functional Nanosystems and High-Temperature Materials, National University of Science and Technology “MISiS”, Leninskiy Prospekt 4, Moscow 119049, Russia; avrore@gmail.com

**Keywords:** metal-carbon nanocomposites, Fe-Co alloy, magnetic nanoparticles, carbon encapsulation

## Abstract

Fe-Co alloy nanoparticles with different sizes, supported by carbon derived from several polymers, namely polyacrylonitrile, polyvinyl alcohol and chitosan, have been synthesized by a one-pot method involving simultaneous metal nanoparticle formation and polymer carbonization. The method involves the joint dissolution of metal salts and a polymer, followed by annealing of the resulting dried film. Detailed XRD analysis confirmed the formation of Fe-Co alloy nanoparticles in each sample, regardless of the initial polymer used. Transmission electron microscopy images showed that the Fe-Co nanoparticles were all spherical, were homogeneously distributed within the carbon support and varied by size depending on the initial polymer nature and synthesis temperature. Fe-Co nanoparticles supported by polyacrylonitrile-derived carbon exhibited the smallest size (6–12 nm), whereas nanoparticles on chitosan-derived carbon support were characterized by the largest particle size (13–38 nm). The size dependence of magnetic properties were studied by a vibrating sample magnetometer at room temperature. For the first time, the critical particle size of Fe-Co alloy nanoparticles with equiatomic composition has been experimentally determined as 13 nm, indicating the transition of magnetic properties from ferromagnetic to superparamagnetic.

## 1. Introduction

Magnetic nanoparticles hold a significant place in modern science and have garnered immense interest due to their exceptional properties, which differ from their bulk counterparts as a result of their reduced size. It is well known that nanomaterials have unique characteristics, which differ from bulk materials due to their larger proportion of surface atoms, resulting in higher surface energy than microparticles or bulk materials [1,2]. Therefore, some metal nanoparticles are pyrophoric and spontaneously ignite in air at room temperature. To address this issue, a common method of protection and stabilization is the creation of a protective shell around the nanoparticles (encapsulation). Carbon is typically utilized as a protective coating for this purpose. The layers of carbon that form on the surface of the metal are usually graphite-like and therefore conductive. Encapsulation of magnetic nanoparticles not only provides protection against oxidation, corrosion and spontaneous aggregation but also helps to maintain their single-domain state [3].

Carbon-supported magnetic nanoparticles of 3d transition metals has gained special interest due to their versatility and extensive application in various fields, such as catalysts for various petrochemical processes [4,5], in medicine for the transport of contrast agents for magnetic resonance imaging (MRI) [6] and for magnetic hyperthermia [7]. They also have potential applications to control the release of drugs in the body [8], as absorbers of electromagnetic waves and in the development of new magnetic materials based on these nanocomposites [9,10].

The combination of metals in a bimetallic system leads to significant improvements in various physicochemical properties. For example, Fe-Co alloy nanoparticles are one of the most popular topics in magnetic nanomaterial research due to their high Curie temperature, low coercivity (H_C_), large saturation magnetization (M_S_), high anisotropy constant and great magnetic permeability [11,12,13]. All of these properties can be fine-tuned by controlling the shape and size of the nanostructures [14,15]. For example, when ferromagnetic particles are reduced to less than a critical size, known as the single-domain state, they transform into superparamagnetic particles, which exhibit high magnetic susceptibility, zero remanent magnetization and zero coercivity.

The single-domain size depends both on various magnetic parameters of the system (exchange parameters, atomic magnetic moment, anisotropy constant), as well as the particle shape, which in turn is determined by the specific synthesis methods employed [16,17]. For Fe nanoparticles, the single-domain size has been estimated to be within the range of 11–30 nm, while for Co nanoparticles, the estimated single-domain size falls within the range of 44–80 nm [16,18,19,20,21,22]. When the nanoparticle size decreases to less than the critical size, the coercivity reduces until the thermal fluctuation of the magnetic moments destroys the existing ferromagnetic order completely. This outcome leads to the nanoparticles becoming superparamagnetic. The coercivity H_C_ of the particles can be roughly correlated with their size, D, per the following relationship [23]:

(1) $$H_{C}=H_{C,(T=0)}\left[1-{\left(\frac{D_{\mathrm{cr}}}{D}\right)}^{3/2}\right]$$

where H_C,(T=0)_ is the particle coercivity at the temperature of 0 K, D is the nanoparticle size, and D_cr_ is the critical nanoparticle size in which the thermal energy k_b_T dominates the anisotropy energy KV, and the magnetic properties change from ferromagnetic to superparamagnetic. 

The magnitude of coercive force is a physical limit that is crucial in determining the potential practical applications of magnetic nanocrystalline materials. For example, superparamagnetic nanoparticles have key importance for biomedical applications because of their tendency to aggregate only in the presence of a strong external magnetic field over long periods of time, whereas ferromagnetic nanoparticles are prone to aggregation even in the absence of an external magnetic field. However, to enhance the efficiency of theranostic applications, such as MRI contrast enhancement and magnetic hyperthermia cancer treatment, magnetic nanoparticles with higher saturation magnetization are required [24]. 

As mentioned above, Fe-Co alloy nanoparticles have been intensively studied because of their excellent magnetic properties, which are extremely dependent on the particle size and synthesis methodology employed. Carbon-supported metal nanoparticles can be fabricated by several methods, including chemical vapor deposition [9,25], chemical reaction methods [26,27], hydrothermal process [28], a pyrolytic route [29], thermal decomposition [30,31] and an arc-discharge method [32]. Nevertheless, the development of simple and inexpensive methods for synthesizing metal nanoparticles with controllable sizes and shapes, as well as high chemical stability against oxidation, remains a challenge.

Previously, a simple one-pot method for the synthesis of metal–carbon nanocomposites based on Fe-Co alloy nanoparticles and carbonized polymers through incoherent infrared (IR) radiation was proposed [33]. IR pyrolysis of the precursor based on a co-solution of polymer and metal salts led to the simultaneous formation of carbon support and bimetallic nanoparticles. The shape and size distribution of the Fe-Co alloy nanoparticles on carbon support can be influenced by the nature of the initial polymer and conditions of synthesis, which in turn can impact their magnetic properties. 

**Figure 1:**
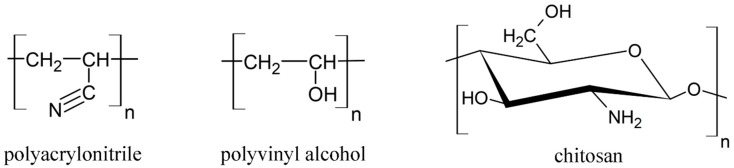
Chemical structures of the polymers used.

In the present paper, we report the synthesis of Fe-Co alloy nanoparticles with different sizes, supported by carbon derived from various pyrolyzed polymers, namely polyacrylonitrile (PAN), polyvinyl alcohol (PVA) and chitosan (CS) (Figure 1), using a one-pot procedure. The structural characteristics and magnetic properties of these nanoparticles have been studied based on their size. For the first time, the particle sizes at which the transition occurs from superparamagnetic to ferromagnetic and from single-domain to multi-domain states were determined for Fe-Co alloy nanoparticles with equiatomic composition.

## 2. Experimental Section

### 2.1. Materials

PAN powder (M_n_ = 73.6 × 10^3^, M_w_= 232.3 × 10^3^) synthesized by the following method [34], PVA (MW = 77–79 kDa, Sigma-Aldrich, Burlington, MA, USA) and CS powder (MW = 500 kDa, deacetylation degree 87%) were purchased from “Bioprogress” (Losino-Petrovsky, Moscow region, Russia) and were employed as carbonaceous precursors. Dimethylformamide C_3_H_7_NO (analytical grade, Acros Organics, Geel, Antwerp, Belgium) and acetic acid (99% purity, Acros Organics, Geel, Antwerp, Belgium) were used as solvents. Iron (III) nitrate nonahydrate (99% purity) and cobalt (II) nitrate hexahydrate (99% purity) were purchased from Acros Organics (Geel, Antwerp, Belgium). 

### 2.2. Nanocomposite Preparation

Two linear and one cyclic polymers were chosen for the synthesis of metal–carbon nanocomposites (Figure 1). These polymers have been extensively studied for their thermal transformations, demonstrating the ability to form carbon at relatively low temperatures (500–600 °C). Moreover, they are manufactured on a large industrial scale, further enhancing their suitability for the intended purpose. 

The procedure for nanocomposite synthesis was the same for each polymer. A polymer and metal compound was dissolved separately in the corresponding solvent. Dimethylformamide and a 2% solution of acetic acid in double distilled water were used as solvents of the PAN and CS powders, respectively. PVA powder was dissolved in double distilled water. The metals were used at an atomic ratio of 1:1 (Fe:Co), and the total amount of metal relative to the initial weight of each polymer was 20%. After complete dissolution of the components, the solutions were mixed, and the co-solution was dried at 80 °C to constant weight. The obtained precursor film was chopped and pre-carbonized at 200 °C for 15 min in air, followed by the main annealing stage at 500, 600 and 700 °C in a nitrogen atmosphere with a heating rate of 50 °C/min and a retention time of 2 min. In the study, a laboratory furnace was used for IR heating [35].

The prepared samples were labeled according to the polymer used: IR-PAN/Fe-Co T, IR-PVA/Fe-Co T and IR-CS/Fe-Co T, where T is a synthesis temperature.

### 2.3. Nanocomposite Characterization

The phase composition and structure of the nanocomposites were determined by an X-ray diffraction (XRD) analysis on a “Difray” 401 powder diffractometer (Scientific Instruments, St. Petersburg, Russia) with Bragg–Brentano focusing. XRD patterns were recorded in the 2θ range of 20–140° using Cr-Kα (wavelength 0.22909 nm) and Co-Kα (wavelength 0.17889) radiations. Co-Kα radiation was used to record more peaks of the Fe-Co solid solution in the XRD pattern for the precise determination of the lattice parameter. The phases were identified using the PDF-2 database of the International Centre for Diffraction Data (ICDD) [36]. The average coherent scattering region (CSR) size was calculated by the Selivanov–Smyslov method from the broadening of the diffraction peaks [37]. The lattice parameters were calculated based on the experimental values of the interplanar spacing using the extrapolation function of Taylor–Sinclair [38].

Transmission electron microscopy (TEM) and selective area electron diffraction (SAED) were performed on JEM-2100 equipment (JEOL, Tokyo, Japan). The particle size measurements from TEM micrographs were performed using ImageJ 1.54d software. OriginPro 2017 software was utilized for the subsequent analysis of the acquired data and the construction of particle size distribution histograms.

The morphology and chemical composition of nanocomposites were studied using scanning electron microscopy (SEM) coupled with energy dispersive X-ray spectroscopy (EDX) on Vega3 SB equipment (TESCAN, Brno, Czech Republic).

Atomic absorption spectroscopy (AAS) of samples was used to determine the actual content of the metal phase and Fe:Co ratio and was performed with an AAnalyst 400 flame atomic absorption spectrometer (Perkin-Elmer, Waltham, MA, USA). Before the analysis, the samples were prepared by burning the carbon support in a muffle furnace at a temperature of 600 °C for 3 h, followed by the dissolution of the metal-containing residue in aqua regia. The relative error of the quantitative content of elements by this method is 5%.

Raman spectra were recorded on a Senterra II (Bruker, Billerica, MA, USA) at a 532-nm wavelength and 0.25 mW of power.

The magnetic characteristics of the nanocomposites were measured by the vibrational magnetometer method [39]. The cell of the vibration magnetometer was a flow quartz microreactor with a volume of 0.3 cm^3^. It was tightly fixed between two membranes of porous quartz. The weight of the magnetite sample was 10 mg in all experiments. Magnetic characteristics of the samples were defined at room temperature via measurements of the magnetization as a function of the applied magnetic field strength. For each sample, three complete cycles of the hysteresis loop were recorded, followed by data averaging.

## 3. Results and Discussion

The series of IR-PAN/Fe-Co, IR-PVA/Fe-Co and IR-CS/Fe-Co nanocomposites were prepared through IR pyrolysis of the precursor based on a co-solution of polymer and metal salts under similar conditions. The key feature of the proposed method is the simultaneous formation of metal nanoparticles and polymer-derived carbon support, resulting in the preparation of metal–carbon nanocomposites. IR pyrolysis of polymer facilitates C-C and C-H bond cleavage reactions led to the formation of carbon and gaseous decomposition products of polymers [34,35]. Therefore, utilizing infrared radiation for heat treatment enables a substantial reduction in the heating time required to reach a desired temperature and exposure time. Polymer decomposition products reduce metal salts to metal nanoparticles, which are then stabilized by carbon. The IR pyrolysis conditions, along with the relative content of metal salts and the nature of the polymers employed, have an effect on the size and structure of carbon-supported metal nanoparticles as a consequence of their properties. Previously, it was shown that the temperature of 500 °C is enough to complete the metal reduction and formation of Fe-Co alloy nanoparticles [33]. In the current study, we compared the impact of the polymers’ nature on the size, structure and magnetic properties of Fe-Co alloy nanoparticles supported by carbon, synthesized at 500, 600 and 700 °C.

Table 1 summarizes the chemical composition of nanocomposites according to atomic absorption spectroscopy and EDX microanalysis, depending on the nature of the polymer and the synthesis temperature of the material. The results indicate that the metal ratio in the carbon support is in line with the expected proportions, and the metal concentration is consistent between the two methods. It can be seen that the actual metal content in the nanocomposites increased with the synthesis temperature. Additionally, the change in the metal content also depends on the nature of the polymer used. Thus, the polymers employed form a series with an increase in the actual metal content of PAN < PVA < CS. Increasing the heating temperature enhances the polymer decomposition, leading to a decrease in the yield of the carbon residue and a relative increase in the concentration of the metal content in the composite. Consequently, this outcome intensifies the coalescence process, resulting in larger metal particle sizes (Table 2). The thermal decomposition behavior of polymers depends on polymer type, sample weight, heating rate and metal type [40,41]. As shown in Table 1, the composites based on pyrolyzed PAN exhibited the highest yield, while those based on CS demonstrated the lowest yield. The observed yield values align with the variation in the actual metal content of the samples.

**Figure 2:**
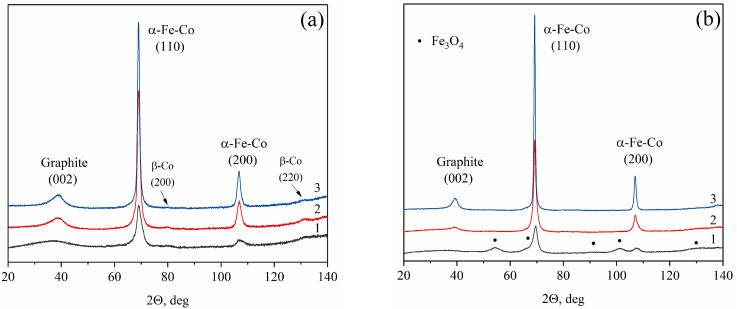

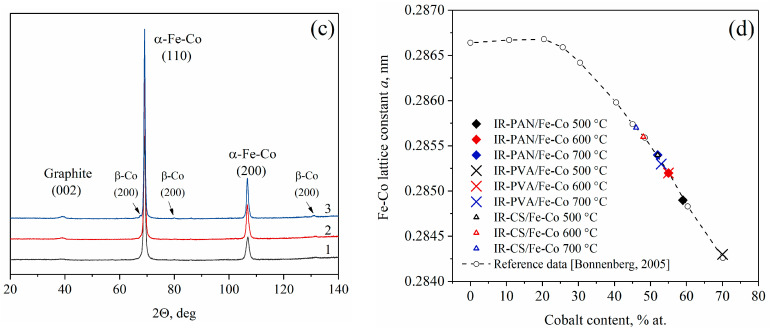
XRD patterns of the IR-PAN/Fe-Co (**a**), IR-PVA/Fe-Co (**b**) and IR-CS/Fe-Co (**c**) nanocomposites, synthesized at 500 °C (1), 600 °C (2) and 700 °C (3). (**d**) The Fe-Co lattice constant change depends on cobalt content according to reference data [Bonnenberg, 2005] and calculated data of the Fe-Co nanoparticles’ lattice constant (color points).

XRD analysis was performed to confirm the successful formation of Fe-Co alloy nanoparticles. Figure 2a–c shows the diffraction peaks corresponding to the crystal planes of (110) and (200), which are attributed to the α-Fe-Co alloy nanoparticles with a body-centered cubic (bcc) structure. Furthermore, the XRD patterns of the IR-PAN/Fe-Co (Figure 2a) and IR-CS/Fe-Co (Figure 2c) samples exhibit additional low-intensity reflections at 2Θ ≈ 67.5°, 80°, and 131°, corresponding to the β-Co phase with a face-centered cubic (fcc) structure. The diffraction peak at 67.5° from the (111) plane is not readily discernible in the XRD patterns of the IR-PAN/Fe-Co samples due to the substantial width of the dominant α-Fe-Co phase. It should be noted that the position of the maxima of this phase is shifted toward smaller angles. For instance, in the case of the IR-CS/Fe-Co 700 °C sample, the interplanar distance of the (111) plane measures 0.2059 nm, whereas according to the PDF-2 database, the interplanar distance for the (111) plane of the β-Co phase is documented as 0.2040 nm. This observation strongly suggests that the face-centered cubic phase also represents a Fe-Co alloy based on the fcc lattice structure of β-Co.

Moreover, additional peaks in the XRD pattern of the sample IR-PVA/Fe-Co 500 °C (Figure 2b), which corresponded to magnetite, were observed. Therefore, a temperature of 500 °C is not sufficient for the complete reduction of metal oxides in nanocomposites based on PVA. The increase in intensity and the narrowing of the Fe-Co diffraction peaks within the range of PAN-, PVA- and CS-employing polymers was observed. This finding can be interpreted as both an increase in the volume fraction of the metal phase relative to the carbon support and an increase in the size of the Fe-Co particles.

Considering the carbon phase of the obtained composites, it should be noted that the samples synthesized at temperatures greater than 600 °C have a diffraction peak at ~39° related to the (002) crystal plane of the graphite phase. This peak indicates the carbon ordering and the formation of a shell with a graphite-like structure around Fe-Co nanoparticles (Figure S1), which possess catalytic ability to convert amorphous carbon into graphite.

The experimentally determined values of interplanar distances, obtained using the Bragg equation, were utilized to calculate the lattice parameters of the Fe-Co alloy nanoparticles using the Taylor–Sinclair extrapolation function [38]. The results are summarized in Table 2 and Figure 2d. In each series of samples, the lattice parameter increases with increased synthesis temperature, indicating a rise in the iron content of the Fe-Co solid solution. This outcome can be explained by both the reduction in residual iron oxide and the formation of a more homogeneous composition of Fe-Co solid solution due to the intensification of coalescence processes at elevated temperatures. The composition of the forming Fe-Co nanoparticles was estimated from the curve of the change in the lattice parameter of the Fe-Co alloy, based on the α-Fe bcc lattice plotted according to the reference data (dash line in Figure 2d). Table 2 shows that the composition of Fe-Co nanoparticles in the nanocomposites based on carbonized PAN and PVA at 500 °C differed the most from the predetermined ratio, especially in the case of the IR-PVA/FeCo 500 °C sample. This finding can be explained by the presence of magnetite phase in the IR-PVA/Fe-Co 500 °C sample. As a result, a cobalt-rich Fe-Co alloy is formed. Additionally, considering the lattice parameter, it can be suggested that a small amount of the iron oxide phase is also present in the IR-PAN/Fe-Co 500 °C sample.

**Figure 3:**
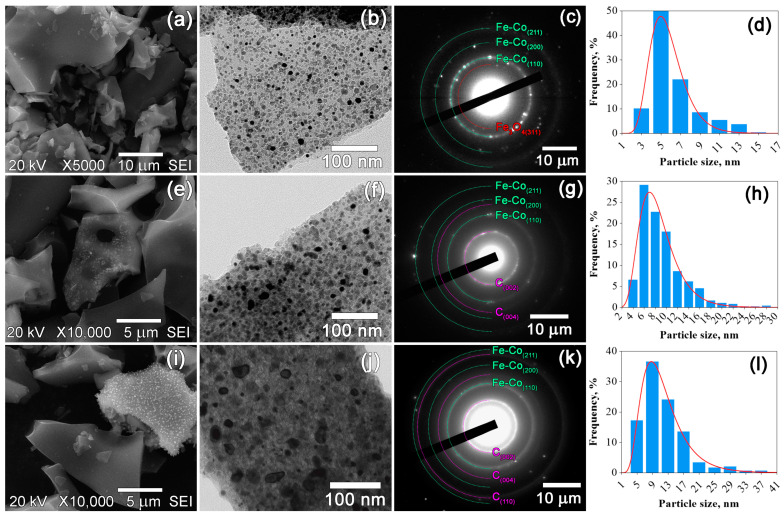
SEM and TEM images, SAED patterns and particle size distributions of IR-PAN/Fe-Co nanocomposites synthesized at 500 °C (**a**–**d**), 600 °C (**e**–**h**) and 700 °C (**i**–**l**).

**Figure 4:**
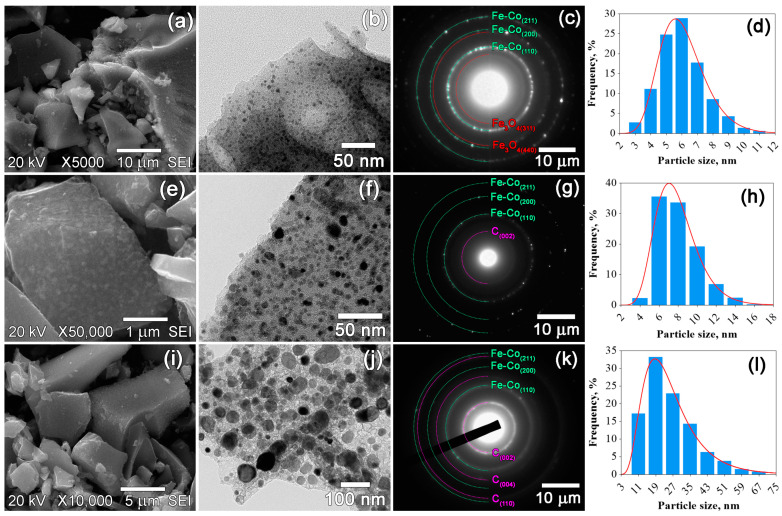
SEM and TEM images, SAED patterns and particle size distributions of IR-PVA/Fe-Co nanocomposites synthesized at 500 °C (**a**–**d**), 600 °C (**e**–**h**) and 700 °C (**i**–**l**).

**Figure 5:**
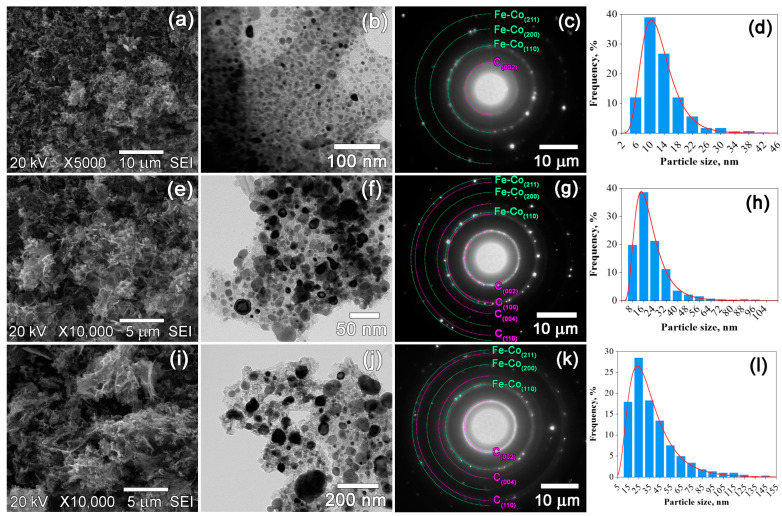
SEM and TEM images, SAED patterns and particle size distributions of IR-CS/Fe-Co nanocomposites synthesized at 500 °C (**a**–**d**), 600 °C (**e**–**h**) and 700 °C (**i**–**l**).

The morphology and particle size distribution of the prepared nanocomposites were assessed by SEM and TEM techniques (Figure 3, Figure 4 and Figure 5). The SEM images revealed notable differences in morphology between the nanocomposites based on CS and those based on PAN and PVA. The morphology of the nanocomposites based on CS is characterized by fracture-damage carbon granules with a sponge-like structure (Figure 5a,e,i). The samples based on PAN and PVA exhibit similar morphologies, characterized by surfaces with fewer pores (Figure 3a,e,i and Figure 4a,e,i). The sponge-like structure is visible only on the fractures of the powder granules. However, as depicted in Figure 3b,f,j, Figure 4b,f,j and Figure 5b,f,j similar structures are observed in all composites. The TEM images clearly represent dark inclusions, indicating the homogeneous distribution of metal nanoparticles within the carbon support. Figure 3c,g,k, Figure 4c,g,k and Figure 5c,g,k show the selected area electron diffraction (SAED) patterns of the prepared nanocomposites, confirming the results of the X-ray phase analysis. In addition to the Fe-Co and graphite phases, the SAED patterns also indicate the presence of a Fe_3_O_4_ phase in the IR-PAN/Fe-Co 500 °C sample, as hypothesized above. The particle size distribution histograms in Figure 3d,h,l, Figure 4d,h,l and Figure 5d,h,l were constructed based on measurements of approximately 1000 nanoparticles from TEM micrographs. The average error in particle size measurements was 0.5 nm. The histograms were successfully described by the lognormal distribution function, indicating an increase in particle size resulting from coalescence processes. As noted above, the average particle size *D*_TEM_ varies depending on the initial polymer used and the synthesis temperature. In the case of IR-PAN/Fe-Co and IR-CS/Fe-Co nanocomposites, a gradual increase in the average particle size ranging from 6 to 12 nm and from 13 to 38 nm within the temperature range of 500–700 °C was observed, respectively. In the case of IR-PVA/Fe-Co samples, the average particle size initially increased insignificantly from 6 to 8 nm within the temperature range of 500–600 °C. However, after heat treatment at 700 °C, the average particle size dramatically increased to 25 nm. The size of coherent scattering regions *D*_CSR_ for Fe-Co nanoparticles correlates with the TEM data, suggesting that the Fe-Co nanoparticles probably consist of a single crystallite (Table 2).

**Figure 6:**
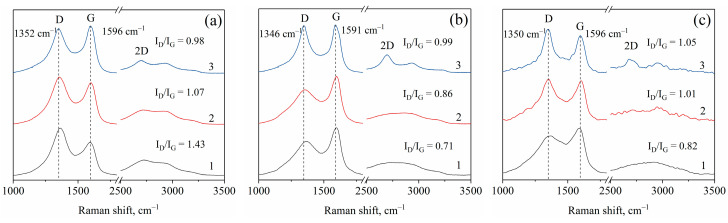
Raman spectra of IR-PAN/Fe-Co (**a**), IR-PVA/Fe-Co (**b**) and IR-CS/Fe-Co (**c**) nanocomposites synthesized at 500 °C (1), 600 °C (2) and 700 °C (3).

To study in detail the carbon support structures derived from various polymers, Raman analysis was performed. In Figure 6, the comparison of the Raman spectra of the synthesized nanocomposites is demonstrated. As shown, all samples exhibited the intrinsic typical carbon features of the D- and G-bands, located at 1346–1352 and 1591–1596 cm^−1^, respectively. The G-band is attributed to all sp^2^ carbons in graphitic sheets and corresponds to the graphitic structure. The D-band signifies the presence of sp^3^ carbon atoms—defective disordered structures. The intensity ratio of D/G peaks serves as a measure of the defects present in the carbon structure. As seen from the Raman spectra, the ratio of I_D_/I_G_ rises as the temperature of calcination increases for the nanocomposites based on PVA and CS, indicating a larger proportion of disordered or oxidized carbon structures. Concurrently, the emergence of a distinct 2D band at ~2700 cm^−1^ and a reduction in the height of the “saddle” around 1500 cm^−1^ signify the formation of ordered planar graphite layers, aligning with the findings of the XRD analysis. In contrast, the intensity ratio of D/G peaks decreases in the case of PAN-based nanocomposites as the calcination temperature increases. This decrease suggests enhanced regularity in the carbon atom arrangement and a reduction in the proportion of defective structures. This distinction can be attributed to the absence of oxygen in the initial structure of PAN. Consequently, the oxidation processes occurring during the pyrolysis of polyacrylonitrile are significantly less pronounced compared to those with polyvinyl alcohol and chitosan.

**Figure 7:**
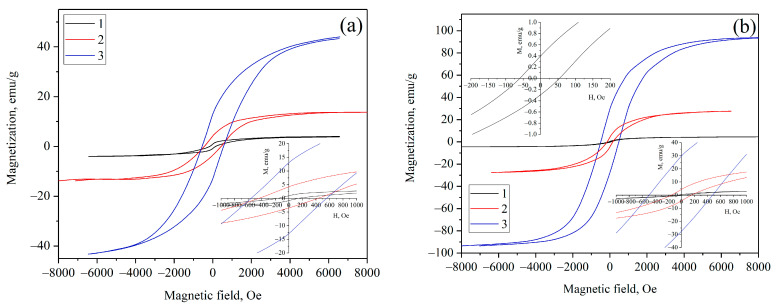

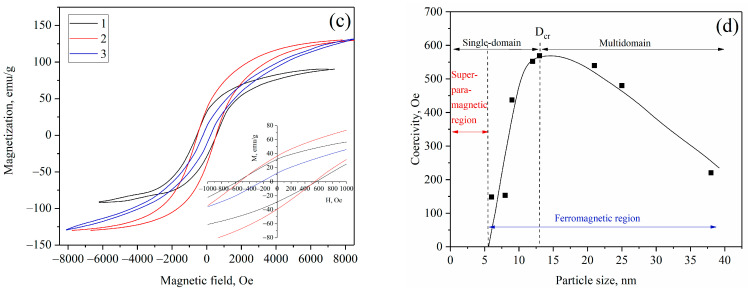
Hysteresis loops of IR-PAN/Fe-Co (**a**), IR-PVA/Fe-Co (**b**) and IR-CS/Fe-Co (**c**) nanocomposites synthesized at 500 °C (1), 600 °C (2) and 700 °C (3). Insets demonstrate enlargement of the hysteresis loops at low fields. (**d**) Coercivity as a function of Fe-Co nanoparticle size (continuous line is the trend).

To investigate the magnetic properties of the Fe-Co alloy nanoparticles, vibrating sample magnetometer analysis was performed at room temperature, and the recorded hysteresis loops are displayed in Figure 7a–c. The insets in the top left and bottom right corners show partial loops in the range of the magnetic field of −1000 and 1000 Oe, respectively. This outcome clearly indicates that nearly all nanocomposites exhibit typical characteristics of hard ferromagnetism, with the coercivity exceeding 125 Oe [43]. The measured properties are listed in Table 3. As expected, M_S_ exhibits a significant increase, ranging from 4.5 to 131.9 emu/g, as the particle size increases from 6 to 38 nm. Additionally, it is observed that the M_S_ value increases with higher calcination temperatures (resulting in larger particle sizes) in each series of composites, based on different polymers. These values are less than those for the bulk Fe-Co alloy (220 emu/g) [44]. However, in our case Fe-Co nanoparticles are incorporated into the carbon support. Hence, the M_S_ value should be corrected, considering the actual metal content in the composite, by Equation (2):

(2) $$M_{S}*=\frac{M_{S}}{\omega }$$

where ω is the actual metal content in the composite according to the AAS data. In this case, the maximum value of M_S_* reaches 200 emu/g, which is already close to the saturation magnetization value of the bulk Fe-Co alloy (Table 3). 

The change in the coercivity with particle size (Figure 7d) was obtained by representing the coercivity value of each sample on a single graph. The IR-PVA/Fe Co 500 °C sample was excluded from Figure 7d to eliminate the contribution of coercive force originating from the magnetite phase, as well as the Fe-Co alloy with a non-equiatomic composition. It was observed that, as the particle size decreases, coercivity increases proportionally to 1/D and attains a maximum at around 13 nm and then decreases abruptly as Hc~D^6^ [45]. The reason lies in the transition to a single-domain state. In the case of multi-domain nanoparticles exhibiting ferromagnetic behavior, the motion of domain walls serves as the primary mechanism for magnetic reversal. Within this size range, the pinning of the domain walls to lattice obstacles, such as grain boundaries, is the main source of the coercivity. As the particle size decreases toward a critical diameter, the system undergoes a transition to the single-domain state. This transition is accompanied by a significant increase in coercivity because the anisotropy energy becomes much greater than the domain wall energy. At this critical particle size, the largest coercivity is typically observed. However, as the particle size continues to decrease beyond this point, the thermal energy starts to become comparable to the anisotropy energy of the particles, and the system becomes superparamagnetic. In the superparamagnetic state, the coercivity decrease is attributed to thermal effects, which are now strong enough to spontaneously demagnetize a previously saturated assembly of particles. According to the plotted dimensional dependence of the coercivity, it can be inferred that the transition between the multidomain and single-domain states is observed at around 13 nm. Furthermore, through extrapolation of the curve to zero, it was determined that the coercivity reaches zero, signifying the transition from a ferromagnetic state to a superparamagnetic state in Fe-Co nanoparticles at an estimated particle size of 5.5 nm.

It should be noted that, in this study, we operated with the average nanoparticle size. Increasing the synthesis temperature of nanocomposites results in both a broadening of the particle size distribution and an increase in the average size, impacting the coercive force and saturation magnetization values. Moreover, a nanocomposite may contain a significant fraction of the superparamagnetic phase, alongside the ferromagnetic phase. This hypothesis is further supported by the low residual magnetization value. 

According to the data in the literature, the single-domain size of Fe_0.65_Co_0.35_ alloy nanoparticles synthesized by the size–control microemulsion method was experimentally estimated to be 9 nm, and the transition from a superparamagnetic to a ferromagnetic state occurred at 4 nm [46]. This paper is the only one we found in which the single-domain size of Fe-Co alloy nanoparticles was experimentally estimated. However, by applying theories of domain stability in fine particles [47] and referencing the available literature on bulk properties, Krishnan et al. estimated the single-domain size of Fe-Co alloy to be 50 nm and the size defined by the superparamagnetic effect to be 18 nm [22]. Our study represents the first experimental determination of the critical particle size for Fe-Co alloy nanoparticles with an equiatomic composition, which is determined to be 13 nm.

## 4. Conclusions

Fe-Co alloy nanoparticles supported by carbon derived from polyacrylonitrile, polyvinyl alcohol and chitosan were prepared by a one-pot method involving simultaneous metal nanoparticle formation and polymer carbonization. The results demonstrated that the choice of polymer precursor and the synthesis temperature had significant impacts on the size, structure and magnetic properties of the Fe-Co alloy nanoparticles and can be utilized for metal-carbon nanocomposites synthesis with a predetermine structure and magnetic properties. The synthesized composites were described as Fe-Co alloy nanoparticles homogeneously distributed within the carbon support, with the metal ratio aligned with the expected proportions. The particle size was primarily determined by the temperature of calcination and the nature of the polymer. It was observed that composites based on pyrolyzed PAN exhibited the smallest Fe-Co particle size (6–12 nm) and the highest yield. In contrast, composites based on CS showed the lowest yield and consequently the largest particle size (13–38 nm). The CS-derived nanocomposites exhibited a sponge-like structure, while those derived from PAN and PVA showed surfaces with fewer pores. Vibrating sample magnetometer analysis indicated that almost all nanocomposites exhibited ferromagnetic behavior, which is typical for hard magnetic material. The observed change in coercivity was attributed to the transition between various size states and, consequently, the magnetization-reversal mechanisms. For the first time, the variations in coercivity nanoparticle sizes at which the transition from a supermagnetic to a ferromagnetic state and from a single-domain to a multi-domain magnetic structure occurs have been defined as 5.5 and 13 nm, respectively.

## Figures and Tables

**Table 1:** Chemical composition and the yield of the metal-carbon nanocomposites.

Sample	Metal Percentage in the Carbon Support *, % wt.		Quantitative EDX Microanalysis, % wt.				Yield, % **
	Fe	Co	Fe	Co	C	O	
IR-PAN/Fe-Co 500 °C	9.0	10.5	7.8	8.5	73.5	10.2	34.1
IR-PAN/Fe-Co 600 °C	11.4	12.5	10.9	11.8	69.4	7.9	27.3
IR-PAN/Fe-Co 700 °C	14.0	14.7	14.5	16.0	63.7	5.8	19.7
IR-PVA/Fe-Co 500 °C	14.8	15.9	15.2	16.0	55.6	13.2	18.9
IR-PVA/Fe-Co 600 °C	17.9	18.3	18.6	19.7	50.2	11.5	17.9
IR-PVA/Fe-Co 700 °C	24.6	25.3	24.0	25.6	44.5	5.9	13.8
IR-CS/Fe-Co 500 °C	30.7	30.3	29.9	31.6	31.0	7.5	10.9
IR-CS/Fe-Co 600 °C	32.0	33.5	31.1	32.2	32.2	4.5	10.6
IR-CS/Fe-Co 700 °C	35.7	34.4	31.7	32.4	32.4	3.5	10.1

* The actual Fe and Co content in the samples according to AAS. ** The yield of the composite relative to initial weight of precursor.

**Table 2:** Structural and dimensional characteristics of Fe-Co nanoparticles.

Sample	Lattice Constant *a*, nm	Fe:Co Ratio, % at.	*D*_TEM_, nm	*D*_CSR_, nm
IR-PAN/Fe-Co 500 °C	0.2849	41:59	6	4
IR-PAN/Fe-Co 600 °C	0.2853	47:53	9	7
IR-PAN/Fe-Co 700 °C	0.2854	48:52	12	12
IR-PVA/Fe-Co 500 °C	0.2843	30:70	6	5
IR-PVA/Fe-Co 600 °C	0.2853	47:53	8	9
IR-PVA/Fe-Co 700 °C	0.2853	47:53	25	24
IR-CS/Fe-Co 500 °C	0.2854	48:52	13	14
IR-CS/Fe-Co 600 °C	0.2856	52:48	21	23
IR-CS/Fe-Co 700 °C	0.2856	52:48	38	37

Lattice constant for α-Fe *a* = 0.2866 nm [42]. *D*_TEM_—the average particle size according to TEM micrographs analysis. *D*_CSR_—the average crystallite size according to XRD analysis.

**Table 3:** Coercivity (H_C_), saturation magnetization (M_S_) and remanent magnetization (M_R_) for metal–carbon nanocomposites at room temperature.

Sample	H_C_, Oe	M_S_, emu/g	M_S_ *, emu/g	M_R_, emu/g	M_R_/M_S_
IR-PAN/Fe-Co 500 °C	148	4.6	23.6	0.9	0.20
IR-PAN/Fe-Co 600 °C	437	13.8	57.7	4.2	0.30
IR-PAN/Fe-Co 700 °C	552	43.9	153.0	13.1	0.33
IR-PVA/Fe-Co 500 °C	57	4.5	14.7	0.4	0.09
IR-PVA/Fe-Co 600 °C	153	27.6	76.2	4.0	0.15
IR-PVA/Fe-Co 700 °C	480	93.8	188.0	31.1	0.33
IR-CS/Fe-Co 500 °C	569	90.4	137.0	32.4	0.37
IR-CS/Fe-Co 600 °C	540	131.3	200.5	37.5	0.28
IR-CS/Fe-Co 700 °C	220	131.9	188.2	12.4	0.09

* The saturation magnetization values in terms of the actual metal content in the composite according to AAS data.

## Data Availability

Not applicable.

## Supplementary Materials

The following supporting information can be downloaded at: https://www.mdpi.com/article/10.3390/ma16206694/s1, Figure S1: HR-TEM micrographs of IR-PAN/FeCo 600 °C (a), IR-PAN/FeCo 700 °C (b), IR-PVA/FeCo 600 °C (c), IR-PVA/FeCo 700 °C (d), IR-CS/FeCo 600 °C (e) and IR-CS/FeCo 700 °C (f) nanocomposites.

## References

[1] Khan Y., Sadia H., Ali Shah S.Z., Khan M.N., Shah A.A., Ullah N., Ullah M.F., Bibi H., Bafakeeh O.T., Khedher N. Ben (2022). Classification, Synthetic, and Characterization Approaches to Nanoparticles, and Their Applications in Various Fields of Nanotechnology: A Review. Catalysts.

[2] Joudeh N., Linke D. (2022). Nanoparticle Classification, Physicochemical Properties, Characterization, and Applications: A Comprehensive Review for Biologists. J. Nanobiotechnol..

[3] Gubin S.P., Koksharov Y.A., Khomutov G.B., Yurkov G.Y. (2005). Magnetic Nanoparticles: Preparation, Structure and Properties. Usp. Khim..

[4] Jia M., Choi C., Wu T.S., Ma C., Kang P., Tao H., Fan Q., Hong S., Liu S., Soo Y.L. (2018). Carbon-Supported Ni Nanoparticles for Efficient CO_2_ Electroreduction. Chem. Sci..

[5] Chernyak S.A., Stolbov D.N., Maslakov K.I., Maksimov S.V., Kazantsev R.V., Eliseev O.L., Moskovskikh D.O., Savilov S.V. (2022). Consolidated Co- and Fe-Based Fischer-Tropsch Catalysts Supported on Jellyfish-like Graphene Nanoflake Framework. Catal. Today.

[6] Bae H., Ahmad T., Rhee I., Chang Y., Jin S.-U., Hong S. (2012). Carbon-Coated Iron Oxide Nanoparticles as Contrast Agents in Magnetic Resonance Imaging. Nanoscale Res. Lett..

[7] Vizcaíno-Anaya L., Herreros-Lucas C., Vila-Fungueiriño J.M., del Carmen Giménez-López M. (2022). Magnetic Hyperthermia Enhancement in Iron-Based Materials Driven by Carbon Support Interactions. Chem. Eur. J..

[8] Hütten A., Sudfeld D., Ennen I., Reiss G., Wojczykowski K., Jutzi P. (2005). Ferromagnetic FeCo Nanoparticles for Biotechnology. J. Magn. Magn. Mater..

[9] Afghahi S.S.S., Shokuhfar A. (2014). Two Step Synthesis, Electromagnetic and Microwave Absorbing Properties of FeCo@C Core-Shell Nanostructure. J. Magn. Magn. Mater..

[10] Ren M., Li F., Wang B., Wei J., Yu Q. (2020). Preparation and Electromagnetic Wave Absorption Properties of Carbon Nanotubes Loaded Fe_3_O_4_ Composites. J. Magn. Magn. Mater..

[11] Antilen Jacob G., Justin Joseyphus R. (2021). Magnetic Properties of FeCo-Iron Oxide Core–Shell Nanoparticles Investigated through First Order Reversal Studies. Appl. Phys. A Mater. Sci. Process..

[12] Karipoth P., Thirumurugan A., Velaga S., Greneche J.M., Justin Joseyphus R. (2016). Magnetic Properties of FeCo Alloy Nanoparticles Synthesized through Instant Chemical Reduction. J. Appl. Phys..

[13] Yang F., Chen H., Liu D., Xiong P., Li W., Chen X. (2017). The Microstructure and Magnetic Properties of FeCo@SiO_2_ Core-Shell Nanoparticles Synthesized by Using a Solution Method. J. Alloys Compd..

[14] Jing P., Du J., Wang J., Zhu Z., Feng H., Liu Z., Liu Q. (2016). Synthesis, Microstructure and Magnetic Performance of FeCo Alloy Nanoribbons. Mater. Lett..

[15] Chokprasombat K., Harding P., Pinitsoontorn S., Maensiri S. (2014). Morphological Alteration and Exceptional Magnetic Properties of Air-Stable FeCo Nanocubes Prepared by a Chemical Reduction Method. J. Magn. Magn. Mater..

[16] Skomski R. (2003). Nanomagnetics. J. Phys. Condens. Matter.

[17] Kamble R.B., Varade V., Ramesh K.P., Prasad V. (2015). Domain Size Correlated Magnetic Properties and Electrical Impedance of Size Dependent Nickel Ferrite Nanoparticles. AIP Adv..

[18] Dormann J.L., Riorani D., Tronc E. (1997). Magnetic Relaxation in Fine-Partilce Systems. Adv. Chem. Phys..

[19] Chen C., Kitakami O., Shimada Y. (1998). Particle Size Effects and Surface Anisotropy in Fe-Based Granular Films. J. Appl. Phys..

[20] Leslie-Pelecky D.L., Rieke R.D. (1996). Magnetic Properties of Nanostructured Materials. Chem. Mater..

[21] Usov N.A., Nesmeyanov M.S. (2020). Multi-Domain Structures in Spheroidal Co Nanoparticles. Sci. Rep..

[22] Krishnan K.M., Pakhomov A.B., Bao Y., Blomqvist P., Chun Y., Gonzales M., Griffin K., Ji X., Roberts B.K. (2006). Nanomagnetism and Spin Electronics: Materials, Microstructure and Novel Properties. J. Mater. Sci..

[23] Frolov G.I., Bachina O.I., Zav’yalova M.M., Ravochkin S.I. (2008). Magnetic Properties of Nanoparticles of 3d Metals. Tech. Phys..

[24] Dave S.R., Gao X. (2009). Monodisperse Magnetic Nanoparticles for Biodetection, Imaging, and Drug Delivery: A Versatile and Evolving Technology. Wiley Interdiscip. Rev. Nanomed. Nanobiotechnol..

[25] Sarno M., Cirillo C., Scudieri C., Polichetti M., Ciambelli P. (2016). Electrochemical Applications of Magnetic Core–Shell Graphene-Coated FeCo Nanoparticles. Ind. Eng. Chem. Res..

[26] Holodelshikov E., Perelshtein I., Gedanken A. (2011). Synthesis of Air Stable FeCo/C Alloy Nanoparticles by Decomposing a Mixture of the Corresponding Metal-Acetyl Acetonates under Their Autogenic Pressure. Inorg. Chem..

[27] Zhang Y., Wang P., Wang Y., Qiao L., Wang T., Li F. (2015). Synthesis and Excellent Electromagnetic Wave Absorption Properties of Parallel Aligned FeCo@C Core-Shell Nanoflake Composites. J. Mater. Chem. C.

[28] Lee S.J., Cho J.H., Lee C., Cho J., Kim Y.R., Park J.K. (2011). Synthesis of Highly Magnetic Graphite-Encapsulated FeCo Nanoparticles Using a Hydrothermal Process. Nanotechnology.

[29] Gupta V., Patra M.K., Shukla A., Saini L., Songara S., Jani R., Vadera S.R., Kumar N. (2014). Synthesis and Investigations on Microwave Absorption Properties of Core-Shell FeCo(C) Alloy Nanoparticles. Sci. Adv. Mater..

[30] Molina J., Valero-Gómez A., Bosch F. (2022). Thermal Synthesis of Pt Nanoparticles on Carbon Paper Supports. Int. J. Hydrog. Energy.

[31] Cervera L., Peréz-Landazábal J.I., Garaio E., Monteserín M., Larumbe S., Martín F., Gómez-Polo C. (2021). Fe-C Nanoparticles Obtained from Thermal Decomposition Employing Sugars as Reducing Agents. J. Alloys Compd..

[32] Han Z., Li D., Wang H., Liu X.G., Li J., Geng D.Y., Zhang Z.D. (2009). Broadband Electromagnetic-Wave Absorption by FeCo/C Nanocapsules. Appl. Phys. Lett..

[33] Vasilev A.A., Efimov M.N., Bondarenko G.N., Muratov D.G., Dzidziguri E.L., Ivantsov M.I., Kulikova M.V., Karpacheva G.P. (2019). Fe-Co Alloy Nanoparticles Supported on IR Pyrolyzed Chitosan as Catalyst for Fischer-Tropsch Synthesis. Chem. Phys. Lett..

[34] Yushkin A.A., Efimov M.N., Vasilev A.A., Bogdanova Y.G., Dolzhikova V.D., Karpacheva G.P., Volkov A.V. (2017). Modification of Polyacrylonitrile Membranes by Incoherent IR Radiation. Pet. Chem..

[35] Efimov M.N., Vasilev A.A., Muratov D.G., Baranchikov A.E., Karpacheva G.P. (2019). IR Radiation Assisted Preparation of KOH-Activated Polymer-Derived Carbon for Methylene Blue Adsorption. J. Environ. Chem. Eng..

[36] PDF-2, The International Centre for Diffraction Data. http://www.icdd.com/index.php/pdf-2/.

[37] Selivanov V.N., Smyslov E.F. (1993). X-ray Diffraction Analysis of the Distribution of Spherical Crystallites in Polydisperse Systems. Crystallogr. Rep..

[38] Taylor A., Sinclair H. (1945). On the Determination of Lattice Parameters by the Debye-Scherrer Method. Proc. Phys. Soc..

[39] Chernavskii P.A., Pankina G.V., Lunin V. (2011). V Magnetometric Methods of Investigation of Supported Catalysts. Russ. Chem. Rev..

[40] Ou C., Li S., Shao J., Fu T., Liu Y., Fan W., Yang X., Bi X. (2016). Effect of Transition Metal Ions on the Thermal Degradation of Chitosan. Cogent Chem..

[41] Lalia-Kantouri M. (2005). Factors Influencing the Thermal Decomposition of Transition Metal Complexes with 2-OH-Aryloximes under Nitrogen. J. Therm. Anal. Calorim..

[42] Bonnenberg D., Hempel K.A., Wijn H.P.J. (2005). 1.2.1.1 Phase Diagrams, Lattice Parameters. 3d 4d 5d Elem. Alloys Compd..

[43] Jiles D.C. (1991). Introduction to Magnetic Materials.

[44] Kuhrt C., Schultz L. (1993). Formation and Magnetic Properties of Nanocrystalline Mechanically Alloyed Fe-Co and Fe-Ni. J. Appl. Phys..

[45] Shokuhfar A., Afghahi S.S.S. (2013). The Heating Effect of Iron-Cobalt Magnetic Nanofluids in an Alternating Magnetic Field: Application in Magnetic Hyperthermia Treatment. Nanoscale Res. Lett..

[46] Shokuhfar A., Afghahi S.S.S. (2014). Size Controlled Synthesis of FeCo Alloy Nanoparticles and Study of the Particle Size and Distribution Effects on Magnetic Properties. Adv. Mater. Sci. Eng..

[47] McKeehan L.W. (1950). Physical Theory of Ferromagnetic Domains. Phys. Rev..

