# Supplementary material for: Fe-Co Alloy Nanoparticles Dispersed in Polymer-Derived Carbon Support: Effect of Initial Polymer Nature on the Size, Structure and Magnetic Properties
Source: Materials (Basel). 2023 Oct 14;16(20):6694. doi: 10.3390/ma16206694 (PMC10608119; doi:10.3390/ma16206694)
Supplement: Supplementary file 1 [file materials-16-06694-s001.zip › materials-2638547-supplementary.pdf]

Fe-Co Alloy Nanoparticles Dispersed in Polymer-Derived Carbon Support: Effect of Initial Polymer Nature on the Size, Structure and Magnetic Properties

Andrey Vasilev [1], Mikhail Efimov [1], Dmitry Muratov [1], Petr Chernavskii [1,2], Kirill Cherednichenko [3], Ella Dzidziguri [4] and Galina Karpacheva [1,*]

[1] A.V. Topchiev Institute of Petrochemical Synthesis RAS, Leninskiy Prospekt 29, Moscow 119991, Russia; vasilev@ips.ac.ru (A.V.); efimov@ips.ac.ru (M.E.); muratov@ips.ac.ru (D.M.); chern5@inbox.ru (P.C.)

[2] Department of Chemistry, Lomonosov Moscow State University, Leninskie Gory 1–3, Moscow 119991, Russia

[3] Department of Physical and Colloidal Chemistry, National University of Oil and Gas "Gubkin University", Leninskiy Prospekt 65, Moscow 119991, Russia; cherednichenko.k@gubkin.ru

[4] Department of Functional Nanosystems and High-Temperature Materials, National University of Science and Technology "MISiS", Leninskiy Prospekt 4, Moscow 119049, Russia; avrore@gmail.com

* Correspondence: gpk@ips.ac.ru

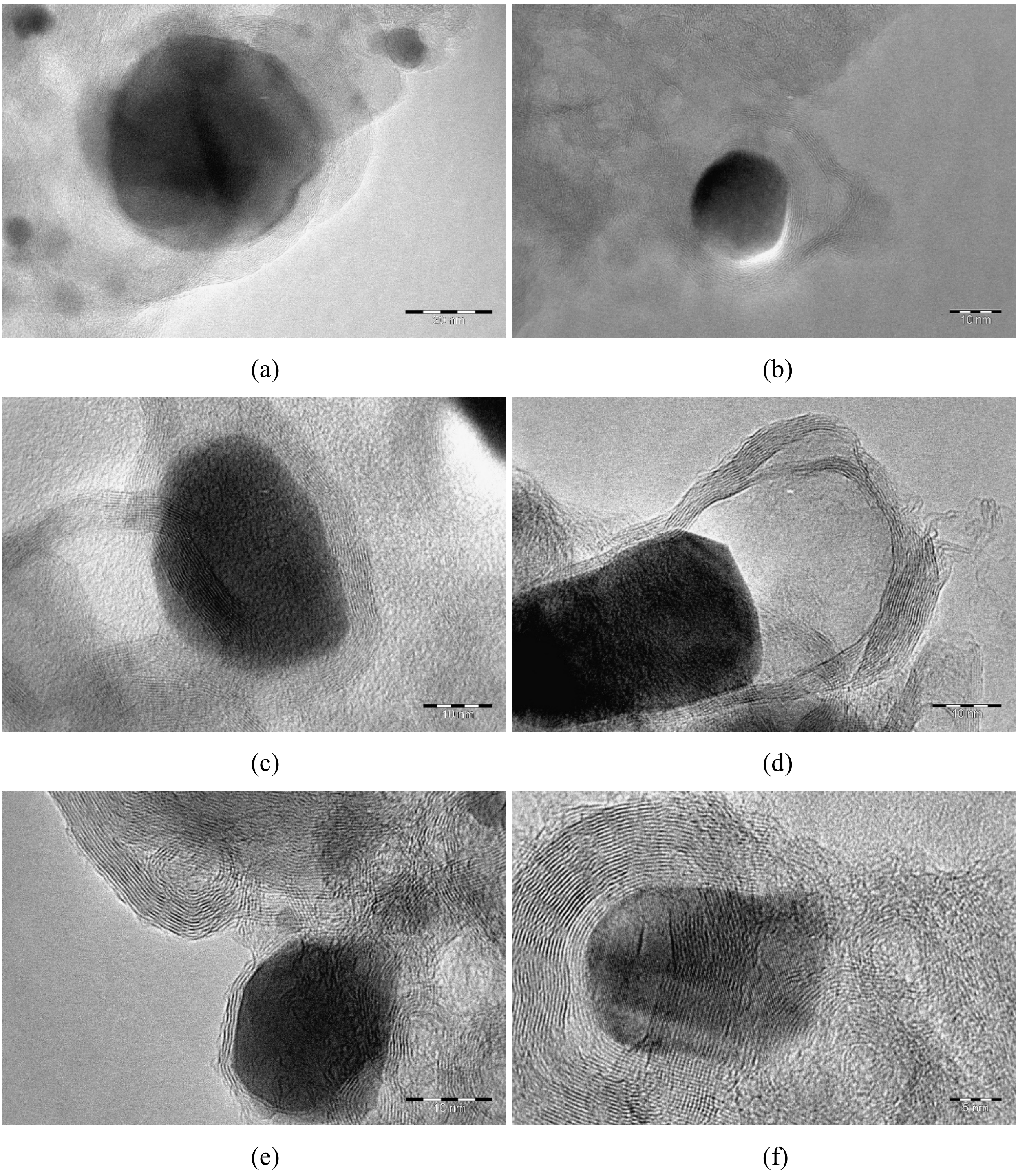

Figure S1. HR-TEM micrographs of IR-PAN/FeCo 600 °C (a), IR-PAN/FeCo 700 °C (b), IR-PVA/FeCo 600 °C (c), IR-PVA/FeCo 700 °C (d), IR-CS/FeCo 600 °C (e) and IR-CS/FeCo 700 °C (f) nanocomposites.
